# The origins of time: A systematic review of the neural signatures of temporal prediction in infancy

**DOI:** 10.1016/j.dcn.2025.101655

**Published:** 2025-12-04

**Authors:** Isabelle Rambosson, Damien Benis, Claire Kabdebon, Didier Grandjean, Manuela Filippa

**Affiliations:** a Neuroscience of Emotion and Affective Dynamics Lab, Department of Psychology, University of Geneva, Geneva, Switzerland; b Swiss Center for Affective Sciences, University of Geneva, Geneva, Switzerland; c CRPN, UMR 7077, CNRS, Aix Marseille Université, France; d Division of Development and Growth, Child and Adolescent Department, University of Geneva, Geneva, Switzerland

**Keywords:** Newborns, Infants, Brain mechanisms, Temporal prediction

## Abstract

Human social interaction relies on the ability to detect and predict the temporal organization of sensory events. Although these abilities change markedly across infancy, little is known about their underlying neural mechanisms. This systematic review aims to define the neural signatures of temporal prediction in newborns and infants and to identify gaps that should guide future longitudinal research. Eight peer-reviewed studies were included, with 228 infants from birth to 9 months of age. Across studies, neural signatures of temporal prediction have been reported in broad cortical areas, including the anterior and medial parts of the brain, particularly within the frontal and central regions. Current evidence suggests that infants' neural responses to temporal regularities likely reflect a combination of early sensory-driven responses and emerging top-down processes. Importantly, gaps in the literature highlight the need for systematic, longitudinal approaches to clarify how neural mechanisms of temporal prediction develop and how biological predispositions and early experiences, including rhythmic and musical interactions, may contribute to this trajectory.

## Highlights

• Temporal predictions are essential in infancy, but evidence remains scattered.
• This review defines temporal prediction neural signatures in neonates and infants.
• Studies showed the frontal and central brain areas' involvement in this process.

## Graphical Abstract

**Figure:**
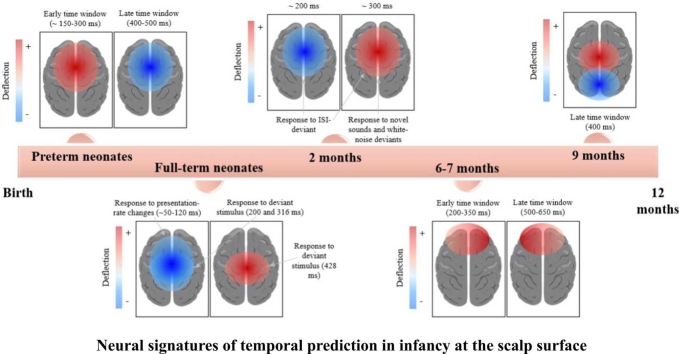
Neural signatures of temporal prediction in infancy at the scalp surface

## 1 Introduction

In the first months of life, young infants undergo a period of intense brain maturation, during which they begin to construct basic knowledge about their environment and the effects of their actions on it. This learning process is not driven solely by bottom-up sensory inputs but also appears to rely on top-down mechanisms, as framed by predictive processing models (Emberson, 2017). Predictive coding, as a general framework, offers a compelling account of how the brain may form and update predictions to interact with a dynamic world (Berger and Posner, 2023, Köster et al., 2020).

Prediction, particularly in its temporal articulation, lies at the intersection of theoretical models, methodological advances, and emerging empirical research in infancy. However, despite growing interest, the organization of the neural mechanisms supporting temporal prediction in early life remains poorly understood. This review, therefore, examines the available evidence on the neural correlates of temporal prediction in newborns and infants while highlighting major gaps in current knowledge.

## 2 Theoretical framework

### 2.1 The predictive brain in adults and infants

Among the theoretical models that have shaped our understanding of infants' cognitive development, predictive coding has emerged as a particularly influential framework, integrating neurodevelopmental, computational, and probabilistic principles. The predictive coding theory (Friston, 2005, Rao and Ballard, 1999) is a unifying brain neuro-computational conceptualization, based on Bayesian inference (Perfors et al., 2011), in which the organism builds probabilistic models, by acquiring statistical regularities from the environment (Clark, 2013) and constructing internal generative models of the world (Friston, 2010, Friston, 2012, Wolpert et al., 1995). There is ample evidence that the adult brain is highly predictive and follows the principles of the predictive coding theory (see Alexander and Brown, 2019, for example; Berger and Posner, 2023, for a review; Carlsson et al., 2000; Kastner et al., 1999; Zhang et al., 2019).

Recent research suggests that a predictive-processing framework, as a broader reference frame, may also provide a unifying perspective for understanding young infants’ cognitive processes (see Köster et al., 2020, for a review), encompassing a range of fundamental knowledge about their social and physical environment, which dynamically coordinates in time. Indeed, in the early phases of development, infants must refine inferences based on sensory inputs—by top-down or feedback neural signals—and minimize prediction errors—through bottom-up processes (Baek et al., 2020). Based on this prediction error related to imprecise or false predictions, the model from which predictions were generated is updated (Berger and Posner, 2023).

These mechanisms, linking past and future inputs, enable our cognitive and affective processes to engage with, react to, and adapt to a constantly changing environment (Baek et al., 2020, Berger and Posner, 2023). Nevertheless, the extent to which these processes operate in infancy—and how they relate to domain-general developmental changes—remains an open question.

### 2.2 Statistical and associative learning as a function and a result of the predictive brain

Complementary to predictive coding, statistical and associative learning provide key mechanisms through which infants acquire structured knowledge about their environment. Statistical learning refers to the ability to implicitly extract and detect recurrent patterns or regularities in sensory inputs—such as in melodies, speech, or visual scenes—without the need for explicit instruction (Aslin and Newport, 2012, Bogaerts et al., 2023, Saffran, 2024). As a core cognitive process embedded within the predictive processing framework, it enables the anticipation of future events by leveraging probabilistic regularities in the environment (Daikoku and Yumoto, 2023, Wang et al., 2025).

During development, infants systematically use statistical learning abilities, for example, to learn about auditory (i.e., linguistics; Saffran, 2020), visual, and proprioceptive inputs (Trainor, 2012). In parallel, associative learning enables infants to form links between distinct stimuli—ranging from concrete sensory events to abstract concepts (Abd El-Hay, 2019, Lafontaine et al., 2020, Mason et al., 2024), through mechanisms shaped by prediction errors (Holland and Schiffino, 2016) and has long been used to investigate fundamental questions about infants’ cognition and learning (Albareda-Castellot et al., 2011, Hochmann et al., 2011, Kovács and Mehler, 2009a, Kovács and Mehler, 2009b, McMurray and Aslin, 2004). These processes may contribute to predictive behavior, although their specificity relative to broader maturational changes is still debated.

### 2.3 Temporal structure and predictions

A key domain where predictive processing has been studied is the temporal structure of sensory input, which enables the brain to anticipate when events are likely to occur. Temporal prediction, or the ability to assess the short-term evolution of forthcoming events in time (Tomasi et al., 2015), relies mostly on implicit basic temporal processing mechanisms such as temporal processing and timing prediction.

The temporal structure of the stimuli in the environment plays a crucial role in infants’ predictive processes, and it constitutes the core element of the present review. Here, we specifically focus on the temporal dimension as the organizing principle of interpersonal coordination. This focus stems from increasing evidence that timing is not merely a supporting element of interaction, but a primary framework through which infants learn to anticipate and respond to the social world.

Indeed, from birth, infants need to extrapolate regularities from the sensory world and coordinate their sensory and motor experience with the physical and human environment (Mento and Granziol, 2020). This coordination process is mainly based on the temporal dimension (Mento and Granziol, 2020). The development of an accurate temporal structure of the inputs/outcomes is critical for the formation of expectations (Mento and Granziol, 2020) in interpersonal synchrony, such as early dyadic and triadic exchanges.

Synchrony, or the temporal coordination of micro-level social behaviors, is based on shared and repetitive rhythmic sequences (Bernieri et al., 1988, Feldman, 2007b). Interindividual synchrony relies on anticipating each other's actions to coordinate in time (Morganti et al., 2008, Sebanz and Knoblich, 2009). Mothers and their infants are involved in interpersonal synchronization from a very early age (Condon and Sander, 1974, Jaffe et al., 2001). By the third month of life, infants actively engage in face-to-face interactions, involving coordinated vocalizations, non-verbal cues, gaze, and shared-emotion expressions (Stern, 1985, Tronick, 1989). Interaction synchrony is fundamental to infants’ cognitive, social, and emotional development (Feldman, 2007a, Jaffe et al., 2001) and relies on anticipatory abilities, which are crucial for the prediction of others’ actions and for producing a coordinated response in time (Crown et al., 2002, Jaffe et al., 2001). A good example of these phenomena is the well-known “peek-a-boo” game in which an adult and an infant engage in violating and confirming time expectations, underpinning infants’ time prediction abilities (Baek et al., 2020).

### 2.4 Methodological approaches used to study prediction

#### 2.4.1 Deviance detection and expectancy violation in adults (ERPs and oscillatory dynamics)

In adults, predictive coding, in particular in the auditory domain, is supported by deviance detection. The latter can be decomposed into two main mechanisms—prediction error and repetition suppression (see Carbajal and Malmierca, 2018, for a review). Deviance detection, which is an automatic response to a divergence (such as a violation) in a stimulation previously processed, allows, in the predictive coding framework, the updating of the prior representational model and favors the integration of this novel information within the previously established framework (Winkler and Schröger, 2015).

In the auditory domain, deviance detection is commonly studied using the auditory oddball paradigm, which consists of introducing a new sound after a series of repeated sounds. Such a sudden shift typically elicits an event-related potential (ERP) known as the mismatch response (MMR) or mismatch negativity, characterized by a frontal positivity synchronized with a posterior negativity occurring 200–400 ms after the deviant stimulus (Basirat et al., 2014, Friederici et al., 2002). Subsequently, this early response is frequently followed 700 ms later by a late frontal Negative Slow Wave (NSW; Basirat et al., 2014; Dehaene-Lambertz and Dehaene, 1994). This ERP response has been hypothesized to originate from neuronal populations over a network comprised of the auditory midbrain and thalamus, displaying stimulus-specific adaptation (SSA, i.e., a modulation of firing rate in response to a deviant stimulus compared to a series of similar stimuli); triggering in this population a reduction over time of the firing response (Carbajal and Malmierca, 2018).

At the same time, considering the oscillatory dynamics related to expectancy violation, a theta synchrony increase has also been observed in a prefrontal subthalamic network in response to error monitoring and expectancy violation (Zavala et al., 2018), and a theta-gamma coupling has been linked to predictive coding in a syllable representation paradigm (Hovsepyan et al., 2020) in adults. In adults, theta band activity has also been shown to consistently decrease as a repeated sequence is learned, in line with SMA predictions (Crivelli-Decker et al., 2018).

#### 2.4.2 Deviance detection and expectancy violation in infants (ERPs and oscillatory dynamics)

Neural mechanisms involved in deviance detection and expectancy violation are already present in newborns (Alho et al., 1990, Bisiacchi et al., 2009, Cheour-Luhtanen et al., 1995, Cheour et al., 1998, Mahmoudzadeh et al., 2017, Mento et al., 2010), as demonstrated by the elicitation of mismatch negativity (MMN) through oddball paradigms, both in newborns (Cheour et al., 2002, Winkler et al., 2003) and during the fetal period (Draganova et al., 2005, Draganova et al., 2007). As shown by Basirat et al. (2014), 3-month-old infants are already able to extract temporal violations, dissociating local—when the stimulus expectancy is induced by a narrow transitional probability—and global violations—when non-adjacent, higher-level rules override local transitions—in an auditory paradigm. Infants process these violations *via* two partially distinct brain systems (i.e., MRR/late frontal negative response for the local deviance condition *vs.* a shorter early mismatch response and a longer late response with cortical sources including the left inferior frontal region in the global deviance condition) (Basirat et al., 2014).

Considering the oscillatory dynamics, findings by Köster et al., 2019, Köster et al., 2021 have shown that unexpected (in contrast to expected) events were linked to theta brain activity in 9-month-old infants, as a key element in the processing of prediction errors, over an extended time window (2 sec) and all the electrodes recorder on the scalp (based on 30 electrodes; Köster et al., 2021).

Findings of theta modulation in infants, as well as accurate interval timing and beat processing within one week of birth in premature (32 weeks gestational age or wGA) and full-term babies (39 wGA) (Edalati et al., 2023, Háden et al., 2024), therefore suggest that adult-like processes are already in place early in human development.

In summary, decades of research have demonstrated that newborns possess neural mechanisms involved in temporal expectancy violations, with the auditory oddball paradigm serving as an effective tool for studying these processes. Recent research has highlighted the importance of theta brain activity in processing prediction errors, emphasizing the complexity and persistence of infants' responses to unexpected events.

#### 2.4.3 Associative learning and anticipatory paradigms

In these experiments, infants are typically taught an association between the presentation of exemplars of a specific auditory or visual (or abstract) category and the subsequent appearance of a reinforcer on the left or right side of the screen. After a familiarization phase, infants (aged from 6 to 12 months) are presented with novel exemplars of the same category, and their ability to correctly anticipate the appearance of the reinforcer on the left or right side of the screen is assessed as a marker for their categorization abilities (see Albareda-Castellot et al., 2011, for example; Hochmann et al., 2011; Kovács and Mehler, 2009a, Kovács and Mehler, 2009b; McMurray and Aslin, 2004).

### 2.5 Relevant literature

#### 2.5.1 Behavioral and physiological studies on (temporal) prediction

##### 2.5.1.1 Prediction

Predictive abilities have been investigated behaviorally in the visual modality in infancy by monitoring looking time after habituation, showing that infants as young as newborns, as well as 2-month-old infants, can extract statistical regularities (Bulf et al., 2011, Kirkham et al., 2002). In addition, by tracking anticipatory eye movements, some studies have demonstrated that, from 3 months of age, infants’ brains can predict the position of a moving object (Canfield and Haith, 1991, Gredebäck and von Hofsten, 2004, Gredebäck et al., 2002) or the end state of a motor action (Ambrosini et al., 2013, Cannon and Woodward, 2012, Falck-Ytter et al., 2006, Kanakogi and Itakura, 2011). Using probabilistic inference in an eye-tracking paradigm, recent work additionally demonstrated that when a situation is uncertain, the brain of 12-month-old infants can spontaneously anticipate the outcome of a scene, demonstrating untrained proactive anticipatory behavior in young infants (Téglás and Bonatti, 2016).

##### 2.5.1.2 Temporal prediction

At a behavioral level, temporal predictions have been investigated in infants with interesting results. In one study, the eye movements of 6- and 12-month-old infants were recorded while observing rational and non-rational feeding actions (Gredebäck and Melinder, 2010). Authors (Gredebäck and Melinder, 2010) reported a change in pupil size—signaling surprise—during violations of infants’ expectations concerning rationality (i.e., in non-rational feeding actions). In another study, Gredebäck et al. (2018) demonstrated that the ability to predict actions in 6-month-old infants is associated with the inclination to react with surprise when social interactions deviate from what was expected. In the auditory modality, Werner et al. (2009) demonstrated using a fixed presentation rate of tone (i.e., fixed condition) and a mixed condition that infants (aged from 6 to 9 months) better detect a tone when it occurred either before or at an expected time following a cue; showing that infants demonstrate greater sensitivity to sounds that align with their temporal predictions.

##### 2.5.1.3 Brain studies on prediction

“Violation”, “prediction”, “expectation”, and “anticipation” are related constructs that reflect a continuum from basic detection to more complex representational and preparatory processes. These terms are often used interchangeably in the literature, creating conceptual ambiguity (Bubic et al., 2010). To clarify this terminology, a violation can be defined as a mismatch between predicted and actual sensory inputs (García Alanis et al., 2023); prediction as a model-based process of forecasting forthcoming inputs (Friston, 2005, Friston, 2010); expectation as a representation, shaped by prior experience, that encodes the most likely outcomes (Bubic et al., 2010); and anticipation as a preparatory or readiness state oriented toward those expected outcomes (Andrzejewski et al., 2019).

Together, these definitions describe a dynamic predictive framework in which cognitive and neural systems continuously generate, refine, and act upon internal models of future events.

Despite this confusion in terminology, the idea of brain mechanisms dedicated to anticipation and prediction has been around for decades, as shown by Piaget’s quotations below:

“*Anticipatory function […] is to be found over and over again, at every level of the cognitive mechanisms and at the very heart of the most elementary habits, even of perception.”*

(Piaget, 1971, p. 191).

“*[…] The function of anticipation is common to cognitive mechanisms at all levels.*”

(Piaget, 1971, p. 193).

If in newborns, dedicated neural mechanisms involved in expectancy violation are already in place (Mahmoudzadeh et al., 2017), it has been much less investigated whether and how early the ability to use this implicit information can be translated into specific, cortical anticipatory processes for future events.

Infants can predict, using, e.g., EEG signals as measures, implying that predictions are inferred from implicit measures, not only the presentation of sequences of stimuli but also the consequences of their actions in the environment (i.e., behavioral inferences). From the age of 2 months, infants consolidate an understanding of the causal link between one of their actions (a foot movement) and the movement of an object linked to it. At 9 months of age, the infant’s brain can, therefore, predict the recurrence of this causal link (Kayhan et al., 2019). Moreover, if the prediction is disregarded (e.g., the object is detached from the foot and no longer moves), infants tend to increase their motor actions to reproduce the coupled event, thereby adapting the predictive model accordingly (Kayhan et al., 2019, Wen and Imamizu, 2022). Interestingly, infants predict an action better when they can perform it, reinforcing the link between action production and perception (Keysers and Perrett, 2004).

Moreover, over the past decade, some research has attempted to identify neural correlates of predictive processing in infancy using associative learning tasks. In 12-month-old infants, Kouider et al. (2015) demonstrated that the acquisition of arbitrary audio-visual associations could modulate visual brain responses, resulting in enhanced EEG activity during early processing stages for expected events and increased neural activity during later stages for unexpected visual events. Subsequent research using similar associative learning tasks also found correlates of increased neural processing in response to expected events in 5-month-old infants (Kabdebon and Dehaene-Lambertz, 2019) and a late difference between expected and unexpected audio-visual associations in 5- to 6-month-old infants (Kabdebon and Dehaene-Lambertz, 2019, Rohlf et al., 2017). Neural signatures of audio-visual associations have also been investigated using EEG in later developmental stages in the context of early word learning within bimodal object-word priming designs. These studies report that consistent object-word priming typically elicits an increase in a frontally distributed N200-500 component and a decrease in the centroparietal N400 component, in 6- to 9-month-old infant ERPs to the word onset (Friedrich and Friederici, 2011, Friedrich et al., 2015, Friedrich et al., 2017), which could be interpreted as cross-modal/semantic prediction effects. Using functional near-infrared spectroscopy (fNIRS), Emberson et al. (2015) presented 6-month-old infants with a systematic audio-visual association, and they were able to record brain activations over the occipital cortex even when the image was unexpectedly omitted after the auditory cue, similar to the response to the actual image presentation. Notably, this occipital activation was not recorded when the auditory cue did not predict any visual event. These results suggest the presence of expectation-based feedback signaling in infancy, although atypical in premature (< 33 wGA) babies (measurements were taken before the infants exhibited clinically identifiable developmental delays at 6 months of corrected age; Emberson, Boldin, et al., 2017), compatible with the predictive coding framework. Finally, a few additional EEG studies from 4 months of age investigated anticipatory pre-stimulus activity in similar audio-visual associative learning tasks in infants, reporting the build-up of a centrally distributed component during the delay between the predictive auditory cue and the onset of the visual stimulus (Kabdebon and Dehaene-Lambertz, 2019, Mento et al., 2022, Mento and Valenza, 2016) comparable to the Contingent Negative Variation (CNV), a neural signature of expectancy-based cortical activity, observed in adult participants.

### 2.6 Adopted perspective and aims of the present review

The perspective adopted in this review assumes that infants can detect certain temporal regularities early in life, yet the neural systems supporting more complex anticipatory or prediction-based processes remain poorly understood. Although early sensory processing and basic regularity detection are observable in newborns, it remains unclear to what extent these responses reflect specialized predictive mechanisms, as opposed to broader maturational or experiential factors.

To date, no comprehensive review has examined the evidence on the neural correlates of temporal prediction in infancy. The present review, therefore, aims to summarize the neural responses reported in studies that explicitly manipulate the temporal structure (or dynamic unfolding) of stimuli, identify major gaps in the current evidence base, and outline methodological considerations needed to guide future longitudinal and mechanistic research.

Clarifying the characteristics and neural substrates of temporal prediction is crucial for advancing our understanding of early cognitive function, which may ultimately support efforts to identify atypical developmental trajectories at an early stage.

## 3 Methods

The search and selection strategies have been conducted following the PRISMA (Preferred Reporting Items for Systematic Reviews and Meta-Analyses) 2020 guidelines (Page et al., 2021).

### 3.1 Literature search strategy

A web-based search strategy was elaborated to identify articles related to the neural signatures of temporal prediction in infancy, including infants from birth to 12 months. To conduct a thorough web-based article search, articles were searched in two electronic databases: CINAHL and Web of Science.

The search was conducted using the keywords mentioned below (Box 1). To find the most plausible possibilities regarding the investigated subject, the terms *Infant* and *Brain* remained fixed in all searches.

Box 1 **Search terms.**

Anticipation, auditory anticipation, auditory violation, baby, brain, cortical, infant, neonate, neural, newborn, prediction, predictive coding, rhythmic violation, sensory prediction, and top-down.

### 3.2 Inclusion and exclusion criteria for the selection of eligible studies

The criteria for inclusion of studies were as follows: (a) published in English, (b) empirical peer-reviewed studies embracing a clear methodological stance, (c) on temporal prediction, (d) 0–12-month-old infants, and (e) with brain outcomes.

Multiple findings of the same article were treated as a single study. Books, book chapters, discussions, theoretical papers, reviews, theses, and dissertations were excluded.

#### 3.2.1 Excluded studies on infants’ prediction: which and why?

The present systematic review aimed to highlight the neural signatures of temporal prediction in infancy. Therefore, we excluded all the papers that did not contain an explicit/strict violation of the temporal structure of a series of regular stimuli. Thus, we excluded the studies employing oddball paradigms that are not based on a rhythmical violation, in which the infant perceives a difference between what is currently presented and what was presented before (e.g., Ackles and Cook, 1998), which translates into terms of deviance or novelty effect during development (Kushnerenko et al., 2013).

In the same vein, we also excluded all the paradigms based on the violation of two paired stimuli (e.g., audio and visual stimuli; see Kersey and Emberson, 2017; Rohlf et al., 2017; Wang et al., 2022, for examples). We excluded studies simply changing the order of the elements in a sequence (e.g., Basirat et al., 2014), or the order of stimulus presentation (e.g., Baek et al., 2022), and studies with repetition suppression paradigms (Emberson et al., 2019, Emberson et al., 2017). In general, it follows that all the studies investigating associative learning paradigms, where the association between two or more stimuli was violated, were excluded.

### 3.3 Results of the search

**Fig. 1:**
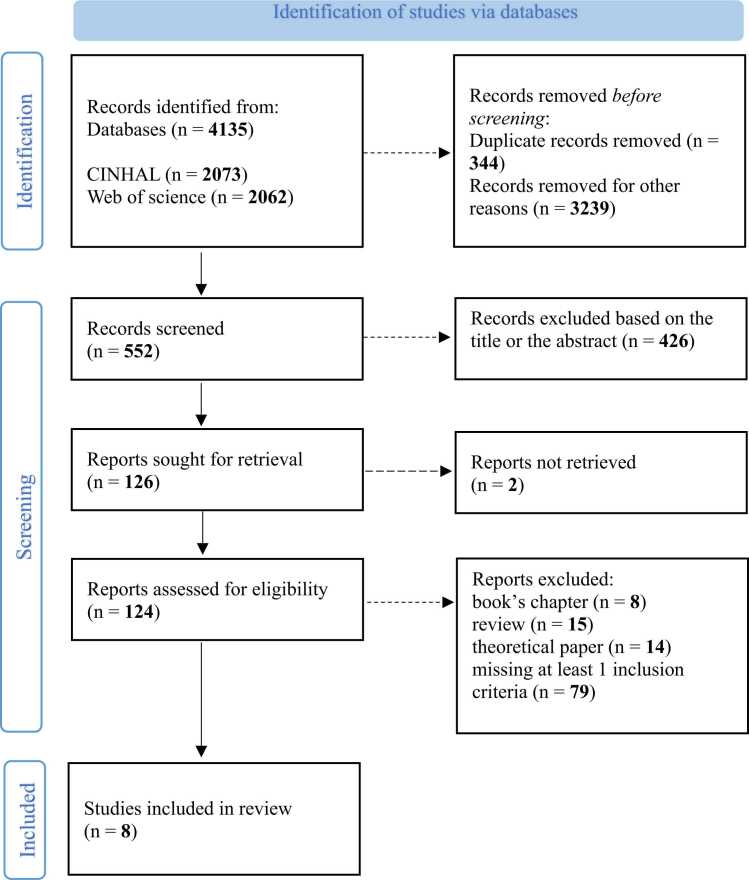
Study selection flow diagram.

The primary literature search identified 4135 potentially relevant references. 426 articles were excluded based solely on the title or abstract. We identified 124 articles for a relevant full-text review. Based on a critical review (using the criteria mentioned in the previous paragraph) of the full text, only 8 articles were identified as eligible for inclusion (cf. Fig. 1).

### 3.4 Data extraction

The principal data were extracted from selected studies and included the author and the year of publication. Studies featured an experimental design or were intervention studies with an experimental design. The experimental paradigm, population characteristics, assessment, and stimuli domains were extracted. Additionally, brain outcome measures, regions of interest, significant results, conclusions, and potential bias assessments were collected from the selected studies.

## 4 Results

### 4.1 Studies’ design and population characteristics

#### 4.1.1 Studies design

All the selected studies had an experimental design, except for one intervention study with a control group (Zhao and Kuhl, 2016). The main characteristics of the studies, along with a brief description of their designs, are presented in Table 1.

**Table 1:** Description of experimental design, population, stimuli domain, and aim.

**Author, reference**	**Experimental design**	**Population** Number, age (mean ± standard deviation; range; sex) Infants’ state at test	**Stimuli domain** Type of stimuli and description	**Aim**
1. Dumont et al. (2022)	Vibrotactile stimulation-omission paradigm: fixed *vs*. jittered ISI (not predictable stimulus onset). Stimuli were interspersed with omission trials: 1 omission trial every 7–12 vibrations.	20 preterm neonates (m at birth = 32.1 ± 0.5 wGA, m at test = 33.3 ± 0.2 corrected wGA; age range at test: 33.0–33.4 corrected wGA; 11 females). Infants were asleep.	Tactile stimuli. Vibration with a palm vibrator, 3 sec. long vibrations interspersed with 5 sec. long intervals. 10 omissions among 84 stimuli. 13 min. total length.	To assess the ability of premature newborns to form a sensory prediction based on a tactile sequence.
2. Edalati et al. (2022)	Auditory rhythmic regularities violations paradigm: oddball paradigm with high-probability standard rhythm (p = 79 %) and infrequent deviant rhythm trials (p = 21 %). Two control sequences without any standard trial in between.	20 preterm neonates (m at birth = 31.48 ± 1.23 wGA; m at test = 33 ± 1.44 wGA; NA; 8 females). Infants were asleep.	Auditory stimuli. Auditory rhythm in 2/4 m, 60 beats/min. Intertone intervals: 1000, 500, 500 ms (standard rhythm), and 1000, 250, 750 ms (deviant rhythm).	To test whether preterm newborns (30–34 wGA) detect temporal regularities in rhythmic patterns. To test the presence of top-down predictions (rhythmic deviations).
3. Háden et al. (2015)	Temporal structure detection paradigm: trains of complex tones varying in pitch (F0) across trains. A pitch was randomly selected for each train. Trains consisted of 8–24 tone repetitions (“slow” rate tones in the 1st part of the train and “fast” rate tones in the 2nd part), followed by a silent interval.	30 full-term neonates (m at birth = 39.7 ± 1 wGA; age range at test: 1–3 days; 14 females). Infants were asleep.	Auditory stimuli. Trains of complex tones with 8 different pitches taken from the C major scale: C3, D3, E3, F3, G3, A3, B3, and C4. Total duration: 22 min.	To evaluate if the newborn brain is sensitive to the overall probability of ascending *vs.* descending pitch steps in the stimulus block at different repetition rates.
4. Maffongelli et al. (2018)	Structural violations of an observed goal-directed action sequences paradigm: control *vs.* violation condition (temporal inversion of two adjacent steps of an action sequence).	14 6–7 months old infants (m = 200 days ± NA; 179–235 days; 7 females). NA	Visual stimuli. Sequences of familiar goal-directed actions are shown as a series of still pictures.	To investigate whether or not a manipulation of the hierarchical organization of an observed familiar action sequence results in differential neurophysiological processing already in infants at the age of 6–7 months.
5. Mento and Valenza (2016)	Audio-visual paradigm: oddball paradigm simulating a “Peekaboo” game. Standard (p = 0.77) *vs.* delayed (p = 0.23) conditions.	15 9 months old infants (m = 283 days ± 23.9; 252–333 days; 5 females). NA 14 adults (m = 25 years ± 3.2; 20–32 years; 9 females).	Visual and auditory stimuli. Visual stimuli: pictures of real female faces, which could be covered by hands or bare-faced and smiling. Auditory stimuli: digitized samples of the notes C_4_, E_4_, G_4_, and C_5_ played by different musical instruments - i.e., trumpet, oboe, saxophone, and piano.	To understand whether (1) anticipatory neural mechanisms instantiated by temporal expectancy are already operating early in life and (2) these rely on similar or different spatiotemporal dynamics as compared to adults.
6. Otte et al. (2013)	Auditory oddball paradigm: violation of temporal regularities. Regular 300 ms ISI between standard tones (p = 0.7). 3 deviants (p = 0.1 each): − 100 ms ISI. − 2 spectral deviants.	76 2 months old infants (m = 70.1 days ± 6.2 days; NA; 46 females). 36 waking (20 females) and 40 sleeping (26 females) infants.	Auditory stimuli. Four types of tones: one standard (300 ms ISI) and three deviants. One deviant, identical to the standard, preceded by a 100 ms ISI. Two spectral deviants (white noise sound and novel sound).	To shed light on whether infants can identify violations of temporal regularities (detection of ISI shortenings). To examine the effects of the state of alertness on the MMR.
7. Winkler et al. (2009)	Meter violation paradigm. Standard patterns: base pattern and 3 standard deviants with omission on the lowest metrical level (p = 22.5 % each). 1 deviant pattern (p = 10 %) with metrically salient omissions in the base pattern. 1 control pattern repeating the deviant pattern 100 % of the time.	14 newborn infants (NA; age range at birth: 37–40 wGA, age range at test: 2–3 days; 3 females). Infants were asleep.	Auditory stimuli. Two-measure rock drum accompaniment pattern composed of snare, bass, and hi-hat spanning 8 equally spaced positions.	To investigate whether newborns can extract the beat of the rhythmical sequence (stronger expectation).
8. Zhao and Kuhl (2016)	Intervention study with experimental design. Auditory oddball paradigm (standard, p = 85 %; deviant, p = 15 %). Effects of a laboratory-controlled music intervention on infants’ neural processing of temporal structure in music and speech: music (intervention group) *vs.* play (control group) activities for 12 sessions.	39 9-months old infants (NA; NA; NA). Intervention group (n = 20) and control group (n = 19). Inclusion in the analysis of the MEG recordings of 36 participants in the music condition (18 from intervention, 6 females; 18 from control, 9 females) and 35 participants in the speech condition (16 from intervention, 4 females; 19 from control, 10 females). NA	Intervention phase: multimodal, social, and repetitive stimuli (synchronization of the infants’ movements to the musical beats in the intervention group *vs.* play without music in the control group). Testing phase: auditory stimuli. Music condition: triple meter structure (strong complex tone combined with two weak complex tones with sound-onset-asynchrony of 300 ms). Speech condition: disyllabic nonword speech (combination of synthesized syllables with silent gaps in between).	To test whether the intervention enhanced infants’ general ability to extract temporal structure and generate more robust predictions about future stimuli in complex auditory sounds.

More specifically, four studies used auditory rhythmic meter, temporal regularities, or goal-directed action structural violations paradigms (Edalati et al., 2022, Maffongelli et al., 2018, Otte et al., 2013, Winkler et al., 2009). Two studies used an auditory or audio-visual paradigm (Mento and Valenza, 2016, Zhao and Kuhl, 2016). The last two studies used a vibrotactile stimulation-omission paradigm (Dumont et al., 2022) and a temporal structure detection paradigm (Háden et al., 2015).

#### 4.1.2 Population

The selected studies included a total population of 228 infants. Among these infants, 40 were preterm (32–33 wGA at birth and 33 wGA at test) neonates (Dumont et al., 2022, Edalati et al., 2022). Two studies were conducted on a total of 44 full-term (39–40 wGA at birth and 1–3 days at test) newborns (Háden et al., 2015, Winkler et al., 2009). One study was conducted on 76 2-month-old participants (Otte et al., 2013). Another study was conducted on 14 6- to 7-month-old infants (Maffongelli et al., 2018). The last two studies recruited 9-month-old infants, comprising a total of 54 infants (Mento and Valenza, 2016, Zhao and Kuhl, 2016).

In four studies, infants were asleep when tested (Dumont et al., 2022, Edalati et al., 2022, Háden et al., 2015, Winkler et al., 2009). In the study by Otte et al. (2013), 36 infants were awake, and 40 were asleep at the time of testing. Nothing has been mentioned about the infant’s state at the moment of the test in the other studies.

One selected study (Mento and Valenza, 2016) has also been conducted on 14 adults. Adults' results have not been included in this systematic review but are presented in the supplementary material (cf. Figures 4 and 5).

### 4.2 Stimuli domains

Five of eight experimental paradigms used auditory stimuli like auditory rhythm, trains of complex tones with different pitches, different types of tones, a two-measure rock drum, or a triple meter structure (Edalati et al., 2022, Háden et al., 2015, Otte et al., 2013, Winkler et al., 2009, Zhao and Kuhl, 2016).

In one study, audio and visual stimuli were paired, where Mento and Valenza (2016) used pictures of real female faces along with digitized samples of notes played by different musical instruments. One study used visual stimuli consisting of sequences of familiar goal-directed actions (Maffongelli et al., 2018). Finally, tactile stimuli have been used only in one study (Dumont et al., 2022).

A more detailed description of the stimuli used in each study is available in Table 1.

### 4.3 Aims

The declared aim of three studies was to assess infants’ abilities to extract temporal regularities in a series of stimuli to form sensory predictions (Dumont et al., 2022, Edalati et al., 2022, Zhao and Kuhl, 2016). Two studies focused on infants’ ability to develop temporal expectations, with a particular interest in anticipatory neuronal mechanisms and their similarity to adults’ spatiotemporal dynamics (Mento and Valenza, 2016, Winkler et al., 2009). The goal of the study by Otte et al. (2013) was to understand whether infants could identify violations in temporal regularities. The last two studies aimed to evaluate infants’ brain sensitivity to the manipulation of pitch steps at different repetition rates (Háden et al., 2015) and the impact of manipulating hierarchical organization on neurological processing (Maffongelli et al., 2018).

### 4.4 Brain outcome measures and regions of interest

#### 4.4.1 Brain outcome measures

Of the eight selected studies, six utilized electroencephalography (EEG) as a measure of brain activity. More information about the type of EEG used, or the number of channels, can be found in Table 2. The measured brain responses were ERPs (e.g., CNV, MMN/MMR; Edalati et al., 2022; Mento and Valenza, 2016; Otte et al., 2013; Winkler et al., 2009). The last two studies used Diffuse Correlation Spectroscopy (DCS) (Dumont et al., 2022) and magnetoencephalography (MEG) (Zhao and Kuhl, 2016) as brain measures.

**Table 2:** Description of brain outcome measures, region of interest, significant results, conclusion, and limitations.

**Author, reference**	**Brain outcome measures**	**Region of interest (ROI)**	**Significant results**	**Conclusion**	**Limitations**
1. Dumont et al. (2022)	Diffuse Correlation Spectroscopy (DCS).	Primary somatosensory cortex.	Jittered ISI: blood flow ➚ during omissions (*t*(8) = 2.622, *p* = 0.031; Cohen’s d = 0.874). Likely but not certain stimulus onset expectation → + somatosensory cortex. Fixed ISI: blood flow ➘ during omissions (*t*(10) = -4.023, *p* = 0.002, Cohen’s d = -1.213). Stimulus onset expectation → − somatosensory cortex.	Sensory prediction is already presented 4 weeks before term. Sensory prediction: opposite somatosensory cortex activity regulation depending on the stimulus onset probability.	High attrition rate (low signal-to-noise ratio of DCS).
2. Edalati et al. (2022)	EEG (124-channel HydroCel Geodesic Sensor Net). − ERPs (MMR).	Frontal and frontocentral regions. Surface EEG and source location.	Deviant rhythm condition: enhanced early (∼150–350 ms) frontal and frontocentral mismatch response (p < 0.05), subsequent negative deflection (400–500 ms). Rhythm violations processing is not limited to primary auditory areas (source location in the DCM analysis): − ⇆ connections between the bilateral A1 and the bilateral STG, the right STG and the right IFG (highest model exceedance probability, *p* = 0.52). Feedback loop within the auditory cortex. The effect size is missing.	Premature neonates detect rhythmic regularities and deviations. Processing of rhythm deviation: primary auditory areas and higher-level temporo-frontal cortical structures in a bottom-up and top-down stream, as in adults. Following deviance detection, an error signal travels forward toward higher-order regions. Then, a feedback loop allows comparison of predicted and sensed stimuli. Onset of the thalamocortical and cortico-cortical circuits: sophisticated structure underlying predictive rhythm processing.	Small population size. The model parameters were those defined by default in DCM in adults.
3. Háden et al. (2015)	EEG (Ag/AgCl electrodes). − ERPs.	Frontal and central regions (electrodes placed at scalp locations F3, Fz, F4, C3, Cz, and C4). Surface EEG.	≠ ERP responses for train onsets, presentation rate changes, and expected tone (train offsets). − Train onset: early negative (0–200 ms, N1) followed by a late positive component (250–400 ms, P2) with a frontocentral maximum. − Presentation-rate changes: early frontocentral negative response (∼50–120 ms) with an apparent central (Cz) maximum. − Expected tone: early distributed positive response (0–150 ms) followed by a negative one (250–350 ms). The effect size is missing.	Newborn’s brain is sensitive to the onset and offset of sound trains as well as changes in the presentation rate. N1 and P2, coding detection and processing of sound, are present in infants during sound-train presentation. At birth, infants are sensitive to temporal aspects of segregating sound sources, speech, and music perception.	NA
4. Maffongelli et al. (2018)	EEG (128-channel Geodesic Sensor Net). − ERPs.	Frontal regions. Surface EEG.	Action sequence structural violation: bilateral anterior positivity in early (200–350 ms) and late (500–650 ms) time windows. Early time window (200–350 ms): + positive amplitude for the violation condition in the right frontal hemisphere (*t*(13) = -2.28, *p* = .01). Late time window (500–650 ms): + positive amplitude for the violation condition in the left frontal hemisphere (*t*(13) = -1.92, *p* = .03). The effect size is missing.	The ability to perceive the temporal structure of familiar actions starts early on in development. Bilateral ERP differences between control and violation conditions in infants (with a significant early response in the right-frontal region).	NA
5. Mento and Valenza (2016)	EEG (geodesic EEG system; high-density 128-channel HydroCel Geodesic Sensor Net). − ERPs (CNV).	Inferior and middle frontal gyri, right temporoparietal areas (inferior and superior parietal cortex, inferior, middle, and superior temporal gyri). Surface EEG and source reconstruction	Central-positive/posterior-negative CNV in infants *vs.* anterior-positive/posterior-negative CNV in adults, soon after the ISI onset to the presentation of the second stimulus (corrected *p* < 0.05). Dynamic changes in anticipatory ERP activity across the task (*p* < 0.05): posterior cluster + negative and anterior/central cluster + positive. − Posterior cluster: ≠ latency ranges. Time-on-task effect 400 ms after the ISI onset in infants *vs.* 170 ms in adults. Brain source reconstruction. Adults and infants: right prefrontal cortex and, in particular, the inferior and the middle frontal gyrus. Infants: temporoparietal areas, with a larger activity in the right hemisphere. Adults: strong activation of the SMA. The effect size is missing.	The human brain’s capacity to translate temporal predictions into anticipatory neural activity emerges early in life, with an adult-like CNV present in infants and adults and common frontal sources in the two populations. The underlying spatiotemporal cortical dynamics change across development, with a temporoparietal shift toward SMA during development.	A small number of artifact-free trials in the delayed condition didn’t allow splitting data into more blocks to test an implicit learning effect.
6. Otte et al. (2013)	EEG (64-electrode locations cap). − ERPs (MMN).	Central, frontal, and parietal regions. Surface EEG.	Frontocentral electrode sites: + amplitude response for all deviants. − ISI-deviant → negative peak (∼200 ms) followed by a smaller late positive wave (starting ∼300 ms). − Novel sounds and white-noise deviant → strong positive wave (peak ∼300 ms). Positive and negative MMRs elicited by ISI-deviant, only in waking infants (*t*_*awake_ISI-deviant*_*(35)* = -3.236, *p* < .01). Amplitudes attenuated in sleeping infants (NA) for white-noise deviant and abolished for ISI-deviant. The state of alertness influences MMR responses: + effect of the state of alertness on ISI-deviant response for the negative MMR. The effect size is missing.	The ability to extract temporal and spectral regularities from a sound sequence is already functional in the first months of life. Infant’s state of alertness → scalp distribution of all deviant-stimulus responses. The ISI-deviant response observed during wakefulness is abolished during sleep.	NA
7. Winkler et al. (2009)	EEG (Ag/Cl-electrodes). − ERPs (MMN).	Central regions (electrodes placed at scalp locations C3, Cz, and C4). Surface EEG.	Brain responses to deviant stimulus ≠ responses to standard and deviant-control patterns (NA). Difference between deviant and deviant-control conditions: negative waves at 200 ms and 316 ms followed by a positive wave at 428 ms (NA). Difference between deviant and standard/deviant-control conditions: significant in 40-ms-long latency ranges centered on the early negative and the late positive difference peaks (NA). Type of stimulus: effect on both peaks (early negative waveform: *F*[2,26] = 3.77, *p* < 0.05, ε = 0.85, and η^2^ = 0.22; positive waveform: *F*[2,26] = 8.26, *p* < 0.01, ε = 0.97, η^2^ = 0.39). Differences between: − deviant and deviant-control responses in both latency ranges (df = 26, *p* < 0.05 and 0.01 for the early negative and the late positive waveforms, respectively). − deviant and standard responses for the positive waveform (df = 26, *p* < 0.01).	Beat violation in a rhythmic sound sequence is detected by newborns and is already functional at birth. Newborns detect regular features in a highly variable acoustic environment. Different responses to a deviant stimulus are associated with the violation of sensory expectations.	NA
8. Zhao and Kuhl (2016)	MEG (306-channel Elekta Neuromag). − MMR.	Temporal and prefrontal areas. Source reconstruction.	Intervention group: − + MMR responses to temporal structure violations around 200 ms in the music condition in both temporal auditory and prefrontal cortical regions (NA). − identical results for temporal structure violations in speech (NA). The effect size is missing.	Music intervention: enhanced neural processing of temporal structure in music and speech, thus enhancing infants’ ability to extract temporal structure information and to predict future events in time.	NA

Abbreviations list of Table 1, Table 2

CNV (contingent negative variation)

DCM (dynamic causal modeling)

EEG (electroencephalography)

ERPs (event-related potentials)

DCS (diffuse correlation spectroscopy)

IFG (inferior frontal gyri)

ISI (interstimulus interval)

MMR (mismatch response)

MMN (mismatch negativity)

SMA (supplementary motor area)

STG (superior temporal gyri)

wGA (weeks gestational age)

#### 4.4.2 Regions of interest

Three studies focused on one specific brain region (Dumont et al., 2022, Maffongelli et al., 2018, Winkler et al., 2009), whereas other studies investigated multiple brain regions.

Most of the selected studies (5/8) concentrated on specific brain activations for temporal prediction in the frontal regions (Edalati et al., 2022, Háden et al., 2015, Maffongelli et al., 2018, Mento and Valenza, 2016, Otte et al., 2013) and three studies in central areas (Háden et al., 2015, Otte et al., 2013, Winkler et al., 2009).

Three studies focused on the involvement of parietal (Otte et al., 2013), temporal (Zhao and Kuhl, 2016), and temporoparietal regions (Mento and Valenza, 2016) in temporal prediction.

One examined specific brain activation related to temporal prediction in frontocentral regions (Edalati et al., 2022), and another focused on prefrontal areas (Zhao and Kuhl, 2016).

The last study involved the primary somatosensory cortex (Dumont et al., 2022).

It’s important to note that six out of eight studies (Edalati et al., 2022, Háden et al., 2015, Maffongelli et al., 2018, Mento and Valenza, 2016, Otte et al., 2013, Winkler et al., 2009) used surface EEG, while Zhao and Kuhl (2016) used source reconstruction in a MEG paradigm. Two studies (Edalati et al., 2022, Mento and Valenza, 2016) used surface EEG along with brain source location or reconstruction. Indeed, in their studies, Edalati et al. (2022) used DCM analysis for brain localization, while Mento and Valenza (2016) identified the regions of interest (i.e., inferior and middle frontal gyri and the right temporoparietal areas) through brain source reconstruction.

More detailed information about brain regions of interest is summarized in Table 2.

### 4.5 Results and conclusions of the included studies

**Fig. 2:**
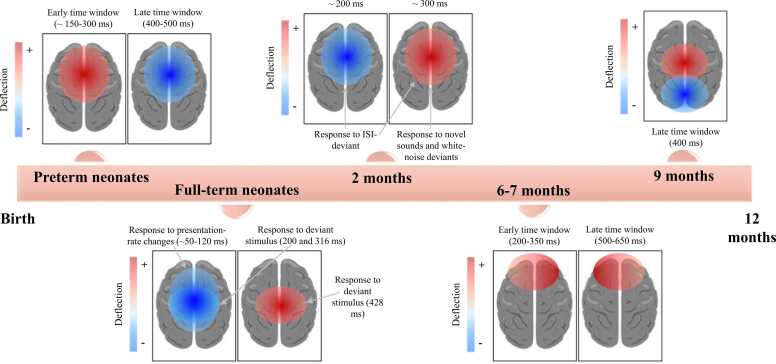
Neural signatures of temporal prediction in infancy at the scalp surface (excerpt from Edalati et al., 2022; Háden et al., 2015; Maffongelli et al., 2018; Mento and Valenza, 2016; Otte et al., 2013; Winkler et al., 2009).

**Fig. 3:**
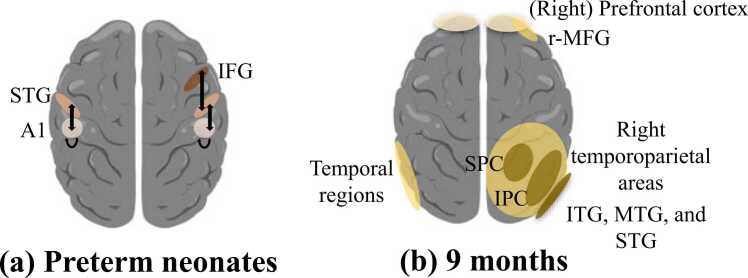
Source location and reconstruction of temporal prediction in (a) preterm neonates and (b) 9-month-old infants (excerpt respectively from Edalati et al, 2022; Mento and Valenza, 2016; Zhao and Kuhl, 2016). (a) In preterm neonates, DCM analysis identified bidirectional connections between the bilateral auditory cortex (A1) and the bilateral superior temporal gyri (STG), the right STG and the right inferior frontal gyrus (IFG), and a feedback loop within the auditory cortex. (b) In 9-month-old infants, source reconstruction identified a cortical activity in the right prefrontal cortex, including the inferior and the middle frontal gyrus (r-MFG) and in the right temporoparietal areas (with an extending activity over the inferior (IPC) and the superior (SPC) parietal cortices, the inferior (ITG), the middle (MTG), and the superior (STG) temporal gyri). Moreover, in 9-month-old infants, a MEG study identified brain activity in the temporal (auditory) and prefrontal cortices.

Results summarizing the main findings of the included studies will be presented from a potential longitudinal perspective (which must be interpreted with caution, cf. limitations of the present review), with the infants’ age groups in Fig. 2, as well as results extracted from source location and reconstruction in Fig. 3.

In preterm neonates aged 33 (corrected) weeks of gestational age, one study showed that in a vibrotactile paradigm, omissions in the jittered interstimulus interval (ISI) condition —where stimulus onset was likely but not certain—induced an activation of the somatosensory cortex, whereas omissions in the fixed ISI condition—where stimulus onset was expected—were associated with deactivation in this region (Dumont et al., 2022). In preterm babies, another study (Edalati et al., 2022) has shown that in an auditory rhythmic regularities violation paradigm, a deviant condition elicited an early (∼150–350 ms) frontal and frontocentral mismatch response (MMR), followed by a negative deflection (400–500 ms). More specifically, using Dynamic Causal Modeling (or DCM) for source location, authors showed that MMR is not limited to the primary auditory cortex but consists of a feedback loop within the auditory cortex, associated with bidirectional connections between the bilateral primary auditory cortex and the bilateral superior temporal gyrus, the right superior temporal gyrus and the right inferior frontal gyrus (Edalati et al., 2022).

In infants born prematurely, sensory prediction following vibrotactile stimulation is already present and involves the primary somatosensory cortex, with an opposite activity regulation depending on the stimulus onset probability (Dumont et al., 2022). Likewise, the infant’s brain extracts rhythmic regularities and detects deviations from them. As in adults, this process also relies on higher-level temporo-frontal brain structures operating in both bottom-up and top-down directions (Edalati et al., 2022). Following deviance detection, an error signal is transmitted to higher-order regions, which in turn send feedback that enables comparison between predicted and perceived stimuli (Edalati et al., 2022).

At birth, a full-term infant’s brain is already sensitive to changes in the rate at which sounds occur, as shown by the elicited early frontocentral negative response (∼50–120 ms) (Háden et al., 2015), and also exhibits distinct neural activity elicited by deviant stimuli (Winkler et al., 2009). Indeed, Winkler et al. (2009) have observed two negative waves (peaking at 200 ms and 316 ms) followed by a positive wave (peaking at 428 ms) in central regions for the difference between the deviant and the deviant control conditions; further post hoc analysis also revealed significant differences between the deviant and the standard response for the positive waveform in the same brain region.

As shown by these studies involving frontal and central regions, the infant brain is already sensitive at birth to the temporal aspects of sounds and their segregation. It can detect regularities in a highly variable acoustic environment, as well as beat violations in a rhythmic sequence that conflict with infants’ sensory expectations (Háden et al., 2015, Winkler et al., 2009).

In 2-month-old infants, an ISI-deviant elicited a negative peak (∼200 ms) followed by a smaller late positive wave (∼300 ms) in the frontal, central, and parietal regions (Otte et al., 2013). In contrast, novel sounds and white-noise deviants elicited a strong positive wave in the same brain regions (Otte et al., 2013). However, the highest-amplitude response has been observed at frontocentral sites for all deviants, although these amplitudes attenuate for white-noise deviants and are abolished for ISI-deviants in sleeping infants (Otte et al., 2013). Notably, Otte et al. (2013) reported that ISI-deviant elicited both positive and negative MMRs only in awake infants, indicating an effect of the infant’s state of alertness (awake *vs.* asleep) on the scalp distributions of MMR responses, with a stronger influence of alertness on the negative-going MMR to ISI deviants.

Early in life, the infant brain can extract temporal regularities from sound sequences. As shown by the authors, the infant’s state of alertness affects the fronto-centro-parietal response to deviant conditions (i.e., the ISI-deviant response observed in waking infants is abolished during sleep) (Otte et al., 2013).

At 6–7 months of age, a structural violation in an action sequence elicited a bilateral anterior positivity in early (200–350 ms) and late (500–650 ms) time windows, with a more positive amplitude for the violation condition in the right and left frontal hemispheres, respectively (Maffongelli et al., 2018).

As demonstrated by this study, 6- to 7-month-old infants can detect the temporal structure of familiar goal-directed actions and their violations, resulting in bilateral ERP modifications in frontal regions (Maffongelli et al., 2018).

At 9 months old, Mento and Valenza (2016) reported anticipatory brain activity, with dynamic changes in anticipatory ERP activity along the task (i.e., the posterior cluster at the surface of the scalp became more negative and the anterior/central cluster more positive indicating, using brain source reconstruction, a larger neural activity in the inferior and middle frontal gyri, and the right temporoparietal areas as the temporal contingency between the two stimuli was implicitly processed by infants’ brain in the delayed condition) in an oddball paradigm simulating a “Peekaboo” game. A laboratory-controlled music intervention (Zhao and Kuhl, 2016) revealed, in both temporal auditory and prefrontal cortical regions (using source reconstruction), larger MMR responses (∼200 ms) to temporal structure violations in music and speech in infants of the same age.

These results indicate that by 9 months of age, the infant brain is already able to translate predictions about temporal sequences into anticipatory activity in prefrontal and temporoparietal regions, resulting in an adult-like CNV (Mento and Valenza, 2016). This neural processing might be enhanced by a music intervention, as shown by Zhao and Kuhl (2016), leading to improved prediction of upcoming events.

More detailed information on the studies’ results is summarized in Table 2.

### 4.6 Bias evaluation

#### 4.6.1 By the authors of their experimental paradigm

Five studies did not report any bias evaluation. Dumont et al. (2022) reported a high attrition rate. Edalati et al. (2022) reported a small sample size (20 preterm neonates in total) and used model parameters based on adults. Mento and Valenza (2016) were unable to test an implicit learning effect due to a small number of artifact-free trials.

## 5 Discussion

The primary objective of this systematic review was to identify the neural signatures of temporal prediction in infancy, to summarize the methodological approaches used to study them, and to highlight key gaps that should guide future longitudinal and mechanistic research.

Across the eight studies included in this review, infants showed measurable neural responses to violations in temporal structure across a variety of auditory and audiovisual paradigms. Although the methods, ages, and analytical approaches varied considerably, several consistent patterns emerged. Over a third of the recruited infants were newborns, demonstrating that age gaps in the existing literature enable the exploration of the emergence of temporal predictive abilities. Within the chosen studies, both premature and full-term infants underwent testing at birth, and at ages 2, 6–7, and 9 months. Notably, there is a testing gap between 2 and 6–7 months, as well as after 9 months, since no studies have examined infants at 12 months of age.

The current systematic review enabled us to summarize the limited evidence available regarding the localization, temporality, and polarity (as measured at the scalp level) of neural responses associated with temporal prediction in infants aged 0–9 months. However, given the small number and methodological heterogeneity of the included studies, these findings cannot be interpreted as evidence of developmental change.

Across studies, infants’ neural responses to temporally structured events were most consistently observed over centro-anterior scalp regions, including the frontal and central areas, although the functional specificity of these activations remains to be clarified in future research.

In preterm, full-term, and 2-month-old infants, bilateral ERP modifications at a similar timing have been observed at the surface of the scalp in fronto-central areas in response to rhythm violation or deviance (Edalati et al., 2022, Háden et al., 2015, Otte et al., 2013, Winkler et al., 2009). These ERP modifications are characterized by an early negative deflection followed by a late positive wave in full-term neonates and 2-month-old infants (Háden et al., 2015, Otte et al., 2013, Winkler et al., 2009); whereas an opposite polarity change is observed in preterm neonates (Edalati et al., 2022). Regarding the temporality of these ERPs in full-term and 2-month-old infants, a common 200-ms negative wave is observed in both paradigms (Otte et al., 2013, Winkler et al., 2009); however, the late positive deflection occurs earlier in the brain of 2-month-old infants (Otte et al., 2013). This earlier response to deviance might indicate, if similar brain processes are involved, a differentiation of deviance detection mechanisms only a few months after birth. The observed polarity and temporality differences between preterm, full-term, and 2-month-old infants (Edalati et al., 2022, Háden et al., 2015, Otte et al., 2013, Winkler et al., 2009) may be explained by the use of different experimental paradigms in each study, as well as different brain mechanisms and/or network involvement, and their neural sources or desynchronization/synchronization.

At 6–7 months of age, bilateral frontal positivity is observed in both early and late time windows in response to structural violations in action sequences (Maffongelli et al., 2018). The difference in observed ERP polarity, compared with the studies mentioned above, could be explained by the nature of the task, which involves action sequences.

Moreover, throughout infants' development, a noticeable shift in activated areas might be observed (measured at the surface of the scalp): central and frontal activity remains highly specific to this early cognitive ability, as observed in studies conducted in 6–7-month- and 9-month-old infants (Maffongelli et al., 2018, Mento and Valenza, 2016), but may extend to posterior areas as they develop (Mento and Valenza, 2016).

Some exceptions in the reported pool of activated brain areas have been noticed (Dumont et al., 2022). This variation can be attributed to the specific paradigm in this DCS study, involving vibrotactile stimulation that elicited distinct somatosensory responses. It is worth noting that we do not posit the primary somatosensory cortex as exclusively dedicated to predictions. Despite the divergence in paradigm design from other studies, this form of stimulation is particularly intriguing and judicious, given that newborns' sensory perception is fundamentally intermodal.

The inquiry into the potential lateralization of prediction mechanisms in infants' brain signatures is interesting. However, the selected studies lack sufficient information to address this question comprehensively, with only two studies providing specificity on lateralization, based on source location and reconstruction, particularly focusing on the right prefrontal cortex and the right temporoparietal regions (Mento and Valenza, 2016), as well as the right STG and right IFG during rhythm violations processing in an auditory paradigm (Edalati et al., 2022).

In one included study (Mento and Valenza, 2016), adults were also tested using the same paradigm as in infants; based on this article, figures presenting the neural signatures at the scalp surface and the source reconstruction of temporal prediction in adults can be found in the supplementary material (cf. Figures 4 and 5).

Some limitations of the included studies emerged, though they did not diminish the significance of their findings. In our view, a term-born control population was missing in Dumont et al. (2022) and Edalati et al. (2022) as a follow-up measure in the first study. Maffongelli et al. (2018) had a limited sample size. In Dumont et al. (2022), brain measurements were limited to the specified region of interest, preventing exploration beyond this area to gain a more comprehensive understanding of whole-brain prediction signatures. Edalati et al. (2022) employed dynamic causal modeling using adults’ model parameters to infer the neuronal architecture underlying the observed mismatch responses in preterm infants.

Moreover, in numerous studies, the infants' state during testing was not specified, posing a limitation to the corpus and, consequently, our conclusions. Studying very young infants is inherently challenging due to their limited periods of wakefulness; however, as they age, the frequency of wakeful periods increases, providing more data on alert infants. The absence of consistent reporting on infants' states during testing or the variability in infants' alertness levels during testing can pose challenges. As noted by Otte et al. (2013), infants’ state of alertness significantly influences brain responses, with sleeping infants showing attenuated amplitudes and only waking infants displaying an ISI-deviant response.

Finally, there is a terminological limitation in the included studies. Terms such as "anticipation", “expectation”, “prediction”, and “violation” are often used without a clear definition, leading to terminological and, therefore, theoretical confusion.

### 5.1 Limitations of the present review and recommendations for further investigations

As a first step towards synthesizing the existing literature on the brain mechanisms of temporal prediction in early ages, this systematic review presents certain methodological limitations that constrain the conclusions that can be drawn from the current evidence base.

First, the focus only on the temporal aspects of prediction (as a subcategory) as a methodological choice of the present review, inevitably restricted the number of articles included, which could potentially limit the impact and generalizability of the present results to all—to some extent, complex and studied using a variety of experimental paradigms—prediction mechanisms. Focusing on the temporal dimension of predictions may offer valuable insights into the core mechanisms underlying certain atypical trajectories—such as those observed in preterm infants, who often exhibit early difficulties in temporal sensory integration and interpersonal synchrony (Feldman, 2007a, Lester et al., 1985).

Second, considering the structural limitations of the current field, the current evidence base is limited by small and scattered samples spanning a wide age range, reflecting the inherent difficulties of studying early developmental stages. Indeed, several studies included small sample sizes, which reduced statistical power and may have potentially led to inflated effect estimates. Moreover, this challenge is compounded by considerable methodological heterogeneity across studies, including variability in experimental paradigms, the selection of brain regions of interest, and heterogeneity in study populations, such as the mixture of preterm and full-term infants or variations in sleep–wake states. Such diversity introduces sources of variance that cannot be disentangled with the available data and likely contribute to inconsistencies in the reported neural signatures of temporal prediction. The use of distinct paradigms—ranging from oddball designs to vibrotactile stimulation and varying in sensory modality, stimulus complexity, probabilistic structure, and violation type—further complicates cross-study comparisons, as these approaches target different aspects of temporal processing. These differences make it difficult to determine whether observed neural responses reflect specific temporal-prediction mechanisms or more domain-general processes, such as basic sensory encoding, processing speed, or habituation. Additionally, all included studies employed cross-sectional designs, making it impossible to draw direct conclusions about developmental change or the maturation of predictive mechanisms over infancy; apparent age-related differences may therefore reflect sampling variability or state-dependent effects rather than true developmental trajectories. Consequently, any developmental perspective suggested in this review must be interpreted with caution, even though predictive abilities appear to be present from very early in life.

Third, a comparison of basal EEG activity measures at the scalp surface (i.e., the morphological characteristics of scalp-recorded waveforms) or brain source location/reconstruction for the concerned studies (Edalati et al., 2022, Mento and Valenza, 2016, Zhao and Kuhl, 2016) from birth to 9 months of age, is challenging and has to be interpreted with caution. Recorded brain activity and the subsequent source location/reconstruction and its comparison across age may be influenced by both maturational (e.g., head size and shape, open fontanelle in neonates, scalp thickness, brain maturation, structural differences such as connectome changes, brain volume conduction, or dipole orientation; Collin and Van Den Heuvel, 2013; Paus, 2024; Reynolds and Richards, 2009) and methodological factors, such as cap montage (especially the reference used; Rosenfeld, 2000). For instance, fixed references (as linked mastoids used in low-density EEG) redistribute electrical activity differently from the average reference commonly used with high-density systems (Chella et al., 2016, Rosenfeld, 2000). While these factors complicate reliable comparisons of scalp distributions and waveform polarity across age groups and tasks, source-level brain analyses can provide significant indicators of the neural generators underlying scalp activity, despite challenges associated with forward modeling and age-specific head models (Michel and Brunet, 2019). In this context, network analysis in preterm neonates from Edalati et al. (2022), as well as the study conduct in 9-months old infants by Mento and Valenza (2016) might be particularly relevant as it report a widespread (pre)fronto-temporo-parietal activation, potentially required the integration of posterior regions (engaged in perceptual processing) with anterior regions (linked to domain general functions) in upcoming studies. Although EEG was the most frequently used technique, studies varied widely in electrode density, reference choices, preprocessing pipelines, and ERP component definitions. This variability complicates comparisons across studies and raises the possibility that differences in reported neural signatures may partly reflect analytical rather than functional distinctions.

Fourth, a subset of studies used alternative imaging modalities (DCS or MEG), each with distinct spatial and temporal characteristics, further increasing methodological heterogeneity.

Collectively, these limitations highlight the need for standardized paradigms, harmonized EEG/MEG analysis pipelines, and longitudinal research designs capable of distinguishing predictive processes from broader maturational changes in early brain function.

Several studies indicate that domain-general processes—such as processing speed, sensory maturation, and basic time perception—play a foundational role in the development of temporal predictive abilities in infancy. For example, visual temporal processing speed (measured by critical flicker fusion thresholds) increases rapidly between 3 and 6 months, approaching adult-like levels by 6 months, suggesting that sensory maturation and processing speed are key contributors to early temporal abilities (Saint et al., 2017). Additionally, multisensory integration, as indexed by the temporal binding window, shows a complex developmental trajectory from infancy through adolescence, further supporting the role of general sensory maturation in temporal processing (Ampollini et al., 2024).

Statistical learning, a domain-general mechanism, is robustly present in infants across both auditory and visual modalities, with evidence that infants as young as 2 months can extract statistical regularities from visual sequences (Kirkham et al., 2002). This supports the idea that infants possess efficient, domain-general learning mechanisms that underpin temporal prediction. While domain-general mechanisms are foundational, research also shows that infants engage in increasingly complex, top-down predictive processing as they mature. Neural signatures of temporal prediction are present from birth and become more sophisticated over the first year, integrating both sensory-driven and higher-order brain regions (Berger and Posner, 2023, Kouider et al., 2015). Recent reviews and meta-analyses suggest that predictive processing in infancy is supported by a large-scale, domain-general neural network, but the expression of temporal prediction may become more domain-specific as cognitive systems mature (Costa et al., 2024, Park et al., 2023). From this perspective, temporal prediction is intrinsically connected to the development of time perception as an early cognitive ability. In speech processing and production, rhythm is closely associated with the development of the speech motor cortex (Poeppel and Assaneo, 2020), shaping the evolution of articulation and rhythm in infant-directed speech. Adults adapt their speech speed to the infant’s perceptual and temporal abilities: from slow and regular to faster and more variable rates (Kitamura and Burnham, 2003, Rosslund et al., 2024). These temporal adjustments help infants acquire predictive models of speech—initially by minimizing prediction errors and later by increasing lexical density, facilitating further vocabulary and syntax acquisition (Cristia, 2013, Weisleder and Fernald, 2013).

Fifth, we recommend that future studies on preterm neonates include a comparison group of typically developing infants to better contextualize neural responses and identify prematurity-related specificities. Indeed, this could enable a more comprehensive comparison and interpretation of the observed effects, providing valuable insights into the developmental trajectory of the studied phenomena and enhancing the generalizability of the findings. Moreover, we recommend meticulously assessing and ensuring uniformity in infants' states during testing to improve the reliability of findings, as variations in behavioral state have been shown to influence neural responses significantly (Otte et al., 2013). Further investigations would also need to confirm the potential effect of music exposure or rhythmical joint activities on neural integration across sensory and higher-order brain regions observed by Zhao and Kuhl (2016) in our corpus.

Finally, future research will require investigating the development and differentiation of predictive brain processes across the first year of life using longitudinal designs. Ideally, these studies would employ homogeneous samples and harmonized methodological approaches, such as a consistent high-density EEG paradigm for temporal deviance detection across different age groups, to reliably map the evolution of temporal prediction mechanisms.

## 6 Conclusion

Current evidence suggests that infants can detect and respond to temporal regularities in their environment, and neurophysiological studies using EEG provide valuable tools for examining these early capacities. While earlier research relying on behavioral observations suggested that predictive abilities are present from birth, neurophysiological investigations have since confirmed and deepened our understanding of these early-emerging capacities. Although these abilities have a broad cerebral localization, most studies indicate that they are concentrated in the anterior and medial parts of the brain, particularly in the frontal and central regions, with consistency across ages. The existing data highlight the possibility that early neural responses to temporal structure reflect a mixture of emerging predictive processes and broader maturational changes in sensory and cognitive function. Experience-dependent factors—such as exposure to rhythmic interactions or musical activities—may also play a role, but current evidence is insufficient to determine their influence. Overall, this review highlights the need for systematic, longitudinal, and mechanistically focused research to clarify how these abilities arise and how they relate to broader cognitive and affective development.

## CRediT authorship contribution statement

**Isabelle Rambosson:** Writing – review & editing, Writing – original draft, Visualization, Methodology, Investigation, Conceptualization. **Damien Benis:** Writing – review & editing, Supervision, Methodology. **Claire Kabdebon:** Writing – original draft, Conceptualization. **Didier Grandjean:** Writing – review & editing, Validation, Supervision, Funding acquisition, Conceptualization. **Manuela Filippa:** Writing – review & editing, Writing – original draft, Validation, Supervision, Methodology, Investigation, Funding acquisition, Conceptualization.

## Funding

This work was supported by the Swiss National Science Foundation [fund number UN11724].

## Declaration of Competing Interest

The authors declare that they have no known competing financial interests or personal relationships that could have appeared to influence the work reported in this paper.

## Data Availability

No new data were created or analyzed in this study. Data sharing does not apply to this systematic review.
