# Supplementary material for: The origins of time: A systematic review of the neural signatures of temporal prediction in infancy
Source: Dev Cogn Neurosci. 2025 Dec 4;77:101655. doi: 10.1016/j.dcn.2025.101655 (PMC12753270; doi:10.1016/j.dcn.2025.101655)
Supplement: Supplementary file 1 — Supplementary material [file mmc1.pdf]

Supplementary material

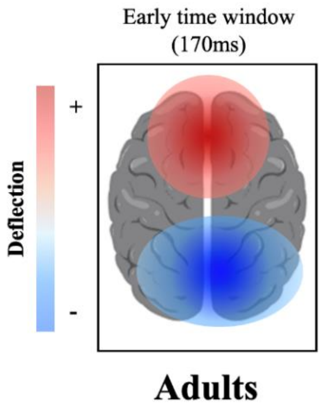

***Figure 4*. Neural signatures of temporal prediction in adults at the scalp surface (excerpt from Mento & Valenza, 2016)**

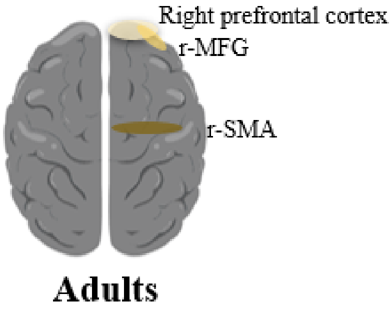

***Figure 5*. Source reconstruction of temporal prediction in adults (excerpt from Mento & Valenza, 2016).** In adults, source reconstruction identified cortical activity in the right prefrontal cortex, including the inferior and the middle frontal gyrus (r-MFG), and in the right supplementary motor area (r-SMA).
